# Progress in Neurology

**Published:** 1898-07-23

**Authors:** 


### Progress in Neurology.

(*Continued from page* 272.)

**Multiple Neuritis.**—Strachan[20] has directed attention to a form of multiple neuritis which is common in the West Indies. A patient suffering from the disease in a typical form complains of numbness and cramps in hands and feet, dimness of vision, and tightness round the waist. There is also a burning sensation in the soles of the feet and palms of the hands, worse at night, and as a rule excoriation and fine desquamation of the edges of the eyelids and margins of lips and nostrils. The main nerve trunks are tender to pressure, especially the ulnar nerve, and along the distribution of the terminal filaments are fine herpetic vesicles. The muscles of the limbs waste, and the trunk muscles may become involved, producing respiratory difficulty. A fatal termination is uncommon. When it does occur, it is preceded by dyspnœa and riotous action of the heart. The mental condition is unimpaired except in the extreme cases in which delusions may be present. The reflexes do not differ in their condition from that found in other kinds of neuritis. In the few necropsies seen the most noteworthy feature seen was pigmentation of the brain, spinal cord, and larger nerve trunks. The changes in the liver and spleen were such as are usually found in cases of malarial poisoning. The author is of opinion that malaria is the most probable toxic feature, though he recognises the possibility that the poison may be the result of some micro-organism or other agency at present unknown.

**Paralysis of the Arm.**—Williamson[21] has recorded a number of cases of paralysis of various muscles of the upper extremity from lesions of the brachial plexus or its branches. Several cases were of a combined paralysis of the deltoid, brachialis anticus, biceps, and supinator longus, a paralysis sometimes known as "Erb's," who first described it. It may be caused in various ways—for instance, by tumour growth (*e g*, a gumma) in the posterior triangle of the neck. It is sometimes met with in newly-born children, where generally the labour has been difficult and some obstetrical operation has been necessary, such as turning and subsequent traction, or energetic pressure of the fingers on the neck. It is often traumatic, produced in many cases by falling with the arm outstretched, or by forcible dragging on the arm. Many cases have been recorded where the paralysis has followed the administration of anæsthetics, chiefly in operations on the breast or axilla, and in these cases the arm has been drawn aside as far as possible upwards and backwards.

Now, the lesion, whether growth or trauma, which produces this combined paralysis, is situated at the junction of the fifth and sixth cervical roots to form the upper cord of the brachial plexus. The mechanism of its injury in cases where the arm has been drawn forcibly upwards is not quite certain; but Williamson finds on dissection that with the arm in this position the upper cord is tightly nipped between the posterior surface of the clavicle and posterior part of the first rib. The nipping is greatest at a point just in front of the articulation of the first rib with the transverse process of the first dorsal vertebra at the posterior part of the rough elevated surface to which the scalenus muscle is attached.

**Facial Paralysis.**—Bordier and Frenkel[22] call attention to a symptom of facial paralysis which was first briefly described by Hilton Fagge. It consists in the fact that when the patient tries to close the eyes whilst looking attentively at an object, the eyelids on the sound side close firmly, whilst on the paralysed side there is very slight narrowing of the palpebral fissure, and the eyeball is turned upward and then slightly outwards. This symptom is marked only when the reaction of degeneration is complete, if the paralysis is less severe, and in recovery it is not found. It is therefore a valuable sign whereby much can be learnt about the progress of the case. The movement of the eyeball is due to the action of the inferior oblique muscle, the nucleus of which has close physiological relations with that portion of the facial nucleus which innervates the orbicularis palpebrarum. Corresponding to the fact that the upper part of the face is but little affected in supranuclear facial paralysis (*e.g.* in hemiplegia), this symptom is not found in that condition.

**Tic.**—Collins[23] in considering this subject says that the word means a twitching. It is applied to an abrupt rapid and usually uncontrollable movement, the result of abnormal contraction of individual muscles or groups of muscles which usually act together to fulfil some physiological purpose. Some have erroneously thought that ordinary tics are simply habits, whilst others have unfortunately regarded tics as atypical manifestations of chorea, and the phrase "a kind of chorea" has been applied to them. The writer considers that tic has nothing in common with chorea. Further, the term, although it suggests only the most prominent symptom, often bespeaks a distinct abnormality of the nervous system. The twitchings to which the term tic applies vary in severity, from the slightest movement of a part up to a most complex co-ordinated action. All tics may be classed primarily into (1) senile (acquired) and (2) early tic, which may be further divided into inherited and acquired. In the majority of cases tic is a degenerative neurosis; on the other hand, simple motor tic, for instance, of the facial muscles is often a reflex condition dependent upon irritation of the branches of the fifth nerve. In regard to treatment, the same principles should guide us as in the treatment of hysteria, epilepsy, and congenital defects in general, and the salts of copper and silver when given in comparatively large doses and for a prolonged time, while the bodily health is cared for, will be found more efficacious than any other therapeutic measure.

**Paræsthesia.**—Dana[24] gives an interesting account of psychrœsthesia or cold sensations. They form a particular class of paræsthesias. There are apparently two classes of cold sensation. In one the symptom is not definitely limited to certain areas, but involves a whole extremity or all four extremities, and is generally associated with other paræsthesias or with pain. In the other class—psychro-æsthesia proper—the sensation is quite an isolated one, and is limited to some special area oftenest upon the thigh or buttock, but sometimes upon the calf or on the face, and more or less closely following the distribution of a nerve. The territory of the external cutaneous nerve of the thigh is a frequent situation for the affection. The sensation is entirely a skin one, and is as if some cold object is lying on the part. The sensation may disappear during warm weather or under exercise. In some instances it is not so much a cold sensation as a cold pain, and it may be obstinate and distressing. This complaint is found more often in men, and almost always in persons over forty years of age. It is caused sometimes by trauma, combined with exposure and a rheumatic tendency. A neuropathic constitution favours its appearance.

A somewhat analogous paræsthesia may be found in syringomyelia, but in that condition the sensation is rather one of general coldness and less sharply limited than in these simple cases. In most cases the treatment is that of an underlying neuritis. Anti-rheumatic drugs, nux vomica, exercise, and electricity are indicated. Locally a liniment containing a little mustard oil is useful. Warm applications and friction sometimes do good. But it must be confessed that in the majority of cases it is not possible to do much to alleviate this distressing condition.

**Neuralgia paræsthetica.**—Sabrazès and Cabannes[25] give an account of this affection. The chief symptom is an abnormal sensation on the antero-external aspect of the thigh in the distribution of the external cutaneous nerve, felt during rest as well as on standing or walking. This sensation may be converted into one of more or less severe pain. Most often the sensation consists of numbness, accompanied or not by formication. Slow, rapid, or prolonged walking may bring on an attack. Usually it is arrested by sitting down for some minutes. The affection is usually unilateral, occasionally bilateral. The paræsthetic zone extends from the anterior superior spine of the ilium to the external condyle of the femur. Sensation is variously affected. This neuralgia is usually a disease by itself, but tabes and diabetes have been noted as causes. Treatment gives variable results. Massage and electricity have been found useful, rest is essential. Resection of the nerve might be advisable in inveterate cases. The affection sometimes undergoes spontaneous cure.

[20] Strachan, Practitioner, Nov., 1897. [21] Williamson, Dec. 11, 1987. [22] Bordier and Frenkel, Sem. Medic., Sept. 9, 1897. [23] Collins, Med. News, Dec. 11, 1897. [24] Dana, New York Med. Jour., Feb. 26, 1897. [25] Sabrazès and Cabannes, Rev. de Méd., Nov., 1897.

